# Wisconsin Medical Society

**Published:** 1875-08

**Authors:** 


### WISCONSIN STATE MEDICAL SOCIETY.

*29th Annual Meeting.*

The 29th Annual Meeting of the Wisconsin State Medical Society opened at the Assembly Chamber, in the Capitol, at Madison, Tuesday evening, June 1st.

Owing, doubtless to the stormy weather yesterday and last evening, there was but a thin attendance.

The meeting was called to order by the President, Dr. J. T. Reeve, of Appleton, at whose invitation, Rev. Thomas Bright, of this city, offered a prayer. Music by the Lake City Band followed, after which the report of the Committee of Arrangements was called for, and in the absence of Dr. Brown, chairman of the committee, Dr. Wm. Fox read the programme of proceedings as fixed upon by the committee. He also added that the members of the Society had been invited by Dr. McDill to visit the Insane Hospital, Wednesday afternoon. He had arranged with the Railroad Company for special trains to carry them to and from the Hospital. Dr. Fox also said that a steamboat excursion upon our beautiful lakes had been arranged by the committee.

On motion of Dr. J. B. Whiting, of Janesville, the report of the committee was adopted.

The Secretary, Dr. Nichols, of Milwaukee, then called the roll of the members of the Society, some fifty answering to their names.

President Reeve then proceeded to address the Society, expressing in the outset his great regret that Dr. Kempster had been unable, on account of sickness in his family, to be present and deliver the lecture which had been expected of him. He tendered his acknowledgments to the Society for having done him the honor to choose him their President, and he hoped the meeting would result in the development of the most friendly feelings among the members. The Society was organized in this city a third of a century ago, and he congratulated its members upon the progress it had made and the good it had accomplished since then. He traced the history of the practice of medical skill from the early ages, when the sick were exposed in the market places, when the priests were the only physicians, and superstitious rites their practice—a state of things that had lasted for 2,000 years, till the time of Esculapius, whose skill in the healing art, it was said, had angered Pluto, robbing that deity of many a subject. Hippocrates had been the first to reduce medical science to anything like order, and to study with cautious care the proper treatment of diseases. He spoke of Galen; of the long period after him when medical science seemed to make no progress; spoke of the later eminent men, such as Servetus, Harvey, Warren and Mott, and declared that medical science did more for human happiness than all the other learned professions combined.

He spoke in condemnation of the class of pretenders and specialists, who claimed to have made new and wonderful discoveries of cures, naming Thessalus, Paracelsus, and, later, one Perkins, who had invented what he called metallic tractors, claiming for them great medical virtues, but which had proved to be worthless. He contrasted these with Hippocrates, who had taken upon himself an oath to study diligently the symptoms of patients, the proper remedies to be administered, and their whole medical treatment. He denied that regular physicians were stationary, as charged by some, but claimed that they were in favor of all right progress; that they stood alone upon medical science and experience, and that their practice embraced the best of eclecticism. He said:

"Our belief and claim is that we stand on the only truly broad and charitable platform—a platform so catholic in its outreaching nature and sympathies, so pure in its purpose to gather from every source whatsoever, all that can be found to contribute to the cure of disease, the prevention of sickness or the prolongation of life, that none seeking the truth need to stray from it, which demands of none blind adherence to specified dogmas, which asks of those who affiliate with us only an intelligent knowledge of their profession, an honesty of purpose in its practice, the keeping in subordination all special dogmas, and the standing alone on professional knowledge and merit for patronage and preferment."

The following are the closing passages of the address:

"To popularize medical knowledge is one of the pressing duties of the day. The field for legitimate medicine in this direction is broad, and too largely uncultivated, yet we rejoice that here and there the good work has been begun. The interests of humanity, the interests of scientific medicine, the thirst of the people for knowledge, all speak to us a lesson—that we give to the people in suitable language pure and healthful medical literature: thus we may save them from many a snare, and from many a destructive pitfall. If we believe our science to be true, let us so present it to the public mind, that its truthfulness will be appreciated, and its practice honored and trusted. If there is aught in it incapable of bearing the closest scrutiny, let that part of it fall, however venerable its usage or however largely supported by authority. Legitimate medicine claims for itself no exclusive privileges; it seeks to conceal nothing, as it needs to conceal nothing; it shrinks from no scrutiny, but ever courts an investigation of its principles and its practice, of its science and its art. In all ages it has been 'the hope of the diseased throughout the world.' Chiefly through its instrumentality have its great hospitals, insane asylums and other charitable institutions of every kind been founded, and to-day it is the custodian of the chief of them throughout the world. So active has it ever been in these works of practical benevolence, breathing the spirit of the Divine Master, Himself the great Physician, that one has termed it Clinical Christianity, and another has spoken of it as Christ's Physical Gospel. Who can estimate its services to the world? Who can compute the value of the discovery of Jenner, which has robbed the world of the terror of one of its greatest pestilences? Who can put a price upon the relief from pain secured by chloroform? How will you estimate the value of life prolonged and sickness prevented? The health of the people is both the wealth and the safety of the nation, and in the preservation and prolongation of this are being achieved, and in the future are yet more to be achieved, some of the 'surest and most glorious triumphs' of medicine.

"Yet great as have been its achievements in the past, there opens up before us a boundless future. The 'Higher Success' of medicine is at once 'the dream' and the ambition of its votaries. The triumphs of medical science in the future are to be bounded only by the intellect and heart which God shall give to its followers, for partaking of the Godlike in its nature and mission, it may reach after the Infinite.

"Let us who are the custodians of its present honor see to it that it passes to the next generation untarnished, ever remembering, in the beautiful language of Dr. Willis, that the servant of religion hath no more of true sanctity about him than the good physician. The service indeed that was rendered of old in special temples to the Divinity, conceived in one of his most beautiful attributes, is not yet extinct upon earth, but has its ministering priest ennobled by Christianity in every worthy member of the profession. Oh, let society cherish its medical community; let it become aware that if science cannot aid in its struggles with disease, neither can ignorance; that nothing can by possibility be known to the quacksalver and ignorant empiric that is not familiar to the educated physician; that a youth of devotion to his art is too little to familiarize him with all the varieties of disease, and the means of meeting them successfully; and that there is no access to the temple of medicine, save through the intimate knowledge of the laws by which we live and move and have our being."

The address was listened to with great attention, and at its close elicited hearty applause. On motion of Dr. Van Dusen, of Mineral Point, the thanks of the Society were unanimously tendered to Dr. Reeve, for his learned, able and interesting address.

Dr. Whiting, of Janesville, inquired of the Secretary if there was any business to present to the meeting; if there was none, he moved to adjourn.

Dr. Marks, of Milwaukee, asked if there was not some unfinished business of the last meeting, which might be acted upon.

The Secretary replied that there was an amendment to the constitution, offered by Dr. Bartlett, last year, which it was proper to act upon. The amendment was to article 9, relating to membership of the Society, and requiring medical students to procure certificates of good moral character and attainments, of the State Board of Censors, and was to confer this power of granting certificates to the censors of county societies, which—after a lively debate, in which Drs. Van Dusen, of Mineral Point, Thomas, of Oshkosh, Whiting, of Janesville, Wight, of Milwaukee, Favill, of Madison, Ferrin, of Mentor, Mason, of Prairie du Chien,, Palmer, of Janesville, Dalton, of Mineral Point, Williams, of Cambridge, and M. M. Davis, of Baraboo, participated—was rejected, by a vote of 22 to 17.

Dr. Van Dusen moved that, as but one of the Board of

Censors, Dr. Mason, was present, Drs. Strong and Bartlett being absent, and there were likely to be candidates for admission to the Society, the President appoint two members to fill the vacancies, and the motion being carried, Drs. Van Dusen and M. M. Davis were so appointed.

The meeting then adjourned to 8 o'clock Wednesday morning.

---

WEDNESDAY, June 2.

The meeting was called to order by the President.

The reports of the Secretary and Treasurer, Dr. F. Nichols, of Milwaukee, of the proceedings of this year, were presented. The receipts of the year were reported to be $566, mainly from membership dues, and the expenditures, of which $220 were for printing transactions, were $387. The reports were adopted, and the Treasurer's report was then referred to the Finance Committee.

Reports of interest to the members of the profession were read as follows:

By Dr. Ira J. Manly, of Markesan, from the Committee on Practice, embodying many interesting cases and suggestions therefrom in regard to treatment of fevers by water, as now practiced extensively in Europe and America. Also in the same paper were remarks on rheumatism and transfusion of blood. The report elicited remarks by Dr. Stoddard, as to whether arterial blood could not be safely infused by the immediate method, instead of venous as heretofore used.

Remarks were made by Dr. Whiting on the use of Peruvian bark in the treatment of rheumatism. Tincture of iron was recommended by Dr. Manly, and alkalies and diaphoretics by Dr. Stansbury. The report was referred to the Committee of Publication.

The report of the Censors recommended the following gentlemen for admission to membership: Dr. James Guinan, New London; Dr. John A. Masterson, Waterloo; Dr. Chas. S. Taylor, Janesville; Dr. J. G. Meachem, Jr., Racine; Dr. Chas. Egan, Highland; Dr. N. H. Norris,

Beloit; Dr. Joseph Van Buskirk, Ironton; Dr. M. R. Gage, Sparta; Dr. L. C. Cowles, Baraboo; Dr. W. H. Vittum, Baraboo; Dr. D. O. Bennett, Waterloo; Dr. D. F. Boughton, Madison; Dr. W. H. Gleason, Mazomanie; Dr. J. J. Leavitt, Fennimore.

The report of the Committee on Surgery was a paper by Dr. Marks, on new methods of ligating arteries. It gave suggestions by Dr. Webber, of Cleveland, of pocketing the extremity of the artery as a substitute for ligation. Dr. M. recommended ligation by deer tendon, carbolized, as a method superior to ligations heretofore used. Discussion ensued on the subject by Drs. Stoddard, Palmer, and others. Dr. Day, of Wauwatosa, gave a case where Dr. Marks used tendonous ligature, and considered it superior to the silk. Dr. Stoddard questioned the reason for applying tendon instead of silk carbolized, both being animal tissues. The report was adopted and referred to the Committee on Publication.

Dr. Whiting moved that papers be referred to the Committee on Publication, after being read in regular order, which was objected to, and rejected by vote.

Some further remarks were made by Dr. Dalton on animal ligatures; also, by President Reeve. Dr. Manly inquired what became of ligatures in the abdominal cavity, which was answered by Dr. Marks, recommending, as before, animal tendons, instead of silk, as being more easily absorbed. This statement was questioned by Drs. Stoddard, Whiting and Palmer. Dr. Palmer sees no superiority in tendon over silk, that also being, as was before stated, an animal substance, and consequently as capable of absorption.

Dr. Faville requested a change of the regular programme, that a committee might be appointed to investigate a case of alleged malpractice. Consented to. A committee of five was chosen by the chair, viz.: Marks, Whiting, Dalton, Mason and Manly—to report immediately.

Dr. Day reported a case of amputation by Dr. Marks, with the use of carbolized deer tendon for ligature, with a very successful result. Had primary union in nearly its full extent, but was retarded somewhat by the necessity of using a silk ligature in one part.

It was voted that the invitation of Dr. McDill to visit the Insane Hospital be accepted.

The Committee on Pathology, Dr. Palmer, begged to be excused, as he had no report prepared—not having been notified.

Dr. Senn read a paper on transplantation of bone, which was adopted and referred as usual.

The Committee on New Remedies presented a paper read by Dr. Armstrong, which elicited considerable discussion. The report was adopted.

A very interesting report of Dr. Wigginton, of the State Hospital for the Insane, on pathology of train diseases, and treatment, was read and referred to the Committee on Publication.

Dr. Palmer reported a case of unusual interest, of tubal pregnancy, and exhibited a post-mortem specimen, to illustrate this condition.

Remarks were made by Dr. McCall on a like case occurring in his practice.

From the Committee on Diseases of the Eye and Ear, Dr. Stoddard read a paper, which was referred to the Committee on Publication.

Adjourned till 1.30 P. M.

AFTERNOON SESSION.

The Society met at 2 P. M. and was called to order by the President.

Dr. Linde read a paper upon Diseases of the Skin, which elicited some discussion and was referred to the Committee on Publication.

Some little time was then occupied in making some arrangements for the banquet in the evening.

Dr. Griffin then read an essay upon small-pox and vaccination in reference to making it compulsory, taking strong ground in favor of such statute law.

The doctor gave statistics from which it would seem that the theory advocated by him should be adopted. After some remarks by members of the Society, the paper was referred to the Committee on Publication.

At about half past three the Society adjourned, and repaired to the North-Western depot where a special train was provided and waiting to take the party around the lake to the Wisconsin Hospital for the Insane. The party was under the escort of Dr. Wiggins, Assistant Physician at the Hospital, and was cordially received at the Asylum by Dr. McDill and his accomplished wife, and by Mrs. Halloday, the long-time faithful matron of the Institution. After looking through the Hospital, where everything was found in first class order, and partaking of choice refreshments generously served, the party returned to the city, and at nine, all assembled at the Park Hotel, where was provided by the Committee of Arrangements a grand banquet.

The large dining hall at the hotel was festooned with flags in a handsome manner and the long rows of tables were burthened with flags, flowers, cakes and delicacies of which no description can do justice.

---

THIRD DAY.

(From the Daily of June 4.)

The State Medical Society met again yesterday morning at 8 o'clock. Several committees were appointed, and a paper of Dr. Kempster was referred to the Committee on Publication.

Dr. H. P. Strong, of Beloit, made a report recommending the following names as suitable persons for officers for the ensuing year, who were elected :

*President*—Dr. J. B. Whiting, Janesville.

*Vice-Presidents*—Dr. Ira Manly, Waukesha, and Dr. F. Senn, Milwaukee.

*Secretary and Treasurer*—Dr. J. T. Reeve, Appleton.

The following were appointed as delegates to the American Medical Association: Drs. Manly, Waterhouse, Strong, Palmer, Butterfield, Fox, Dodson, Mason, Armstrong, Rogers, Stansbury, Bond, Griffin, Cory, Zeilley, Britt, Linde, Whiting, Smith, and J. Hobbins.

The following resolution expressing disapprobation of President Bascom's remarks on the subject of mixed medical schools in connection with the State University, was adopted:

*Resolved*, That the Society express its disapprobation of any action looking to any mixed medical schools in connection with the State University.

A vote of thanks was extended to President Reeve and Secretary Nichols, of the Society, for the prompt manner in which they had performed their duties; also to M. H. Irish, of the Park Hotel, for his courteous efforts to make them and their families feel at home, and for the banquet provided; also to the Press for the interest manifested in their meeting; also to Dr. McDill, of the Hospital for the Insane, for kindness shown them; also to Col. Knight, Superintendent of Public Property, for the use of his room.

The Society voted to hold its next meeting in Milwaukee, and the Committee of Arrangements were instructed to prepare a banquet.

Adjourned.

---

